# Impact of sleep patterns upon female neuroendocrinology and reproductive outcomes: a comprehensive review

**DOI:** 10.1186/s12958-022-00889-3

**Published:** 2022-01-18

**Authors:** Gabriela Beroukhim, Ecem Esencan, David B. Seifer

**Affiliations:** Department of Obstetrics, Gynecology, and Reproductive Sciences at Yale School of Medicine, 333 Cedar St, New Haven, CT 06510 USA

**Keywords:** Sleep, Sleep disturbance, Circadian rhythm, Reproduction, Reproductive hormones, Fertility, Infertility, Neuroendocrinology

## Abstract

Sleep is vital to human bodily function. Growing evidence indicates that sleep deprivation, disruption, dysrhythmia, and disorders are associated with impaired reproductive function and poor clinical outcomes in women. These associations are largely mediated by molecular-genetic and hormonal pathways, which are crucial for the complex and time sensitive processes of hormone synthesis/secretion, folliculogenesis, ovulation, fertilization, implantation, and menstruation. Pathologic sleep patterns are closely linked to menstrual irregularity, polycystic ovarian syndrome, premature ovarian insufficiency, sub/infertility, and early pregnancy loss. Measures of success with assisted reproductive technology are also lower among women who engage in shift work, or experience sleep disruption or short sleep duration. Extremes of sleep duration, poor sleep quality, sleep disordered breathing, and shift work are also associated with several harmful conditions in pregnancy, including gestational diabetes and hypertensive disorders. While accumulating evidence implicates pathologic sleep patterns in impaired reproductive function and poor reproductive outcomes, additional research is needed to determine causality and propose therapeutic interventions.

## Backround

While the purpose of sleep remains largely unknown, the importance of sleep is undeniable. One third of human life is devoted to sleep [1]. Sleep is essential to physical health [1], cognitive function [2–4], and mental wellbeing [5]. Yet, one of three US adults report getting less than the recommended seven hours of sleep a night [1]. Inadequate sleep is associated with many chronic diseases and conditions, such as asthma, stroke, coronary artery disease, myocardial infarction, arthritis, diabetes, obesity, chronic obstructive pulmonary disease (COPD), chronic kidney disease, and depression [1, 6].

Growing evidence indicates that sleep is also essential to reproductive function. Importantly, sleep may be a significant modifiable target to improve reproductive health and outcomes. Rising rates of infertility over the last several decades parallel the increasing prevalence of sleep deprivation and disruption [6]. Average sleep duration is reported to be 6.8 h nightly, compared to nine hours observed a century ago, a shift that can in part be attributed to societal and work-related trends [6–8]. Approximately one in three infertile women report disturbed sleep [9, 10]; and one in three infertile women report poor sleep quality [11]. This association, however, may be bidirectional––that is disturbed sleep may interfere with fertility and/or the stress associated with infertility may contribute to poor sleep quality and duration. Moreover, short sleep time and impaired sleep parameters are associated with low socioeconomic status [12], sedentary time [13], and other potential confounders, making it challenging to ascertain whether associations are causal or confounded.

Healthy sleep consists of recurring cycles of 90–120 min, comprising four to six phases of alternating rapid eye movement (REM) and non–REM sleep [14, 15]. Potentially relevant parameters of sleep include duration, chronotype, quality, fragmentation, hypoxia, circadian dysrhythmia, and pathologies, such as apnea or narcolepsy. In humans, sleep is more commonly studied via questionnaires. Occasionally, studies report objective measures of sleep via polysomnography, which combines electroencephalography, electrooculography, and electromyography, alongside recordings of heart rate, respiratory rate, and leg movements as well as actigraphy systems [16].

Several relatively recent review articles have offered compelling clinical evidence for the association between sleep patterns and fertility and stressed the need for further study [6, 16, 17]. In this comprehensive review, we examine sleep-related molecular genetic and endocrinologic mechanisms affecting reproductive physiology. We also present meaningful associations between sleep patterns and parameters of female reproductive health, capacity, and outcomes, rather than focusing solely on fertility/infertility.

The primary purpose of this review is to better understand the potential mechanisms by which altered sleep patterns may impact female reproductive function and assess a variety of clinical reproductive sequelae of sleep alterations. Our specific aims are threefold: (1) review the molecular genetic and endocrinologic mechanisms underlying sleep-related changes in female reproductive processes, (2) explore the effects of sleep and sleep-related endocrinopathies on the reproductive health of women, including ovarian dysfunction (menstrual irregularity, polycystic ovarian syndrome (PCOS), premature ovarian insufficiency (POI), natural fertility, outcomes of assisted reproductive technology (ART), and pregnancy outcomes, (3) assess the available evidence on whether modifying sleep parameters may improve endocrinopathies and reproductive outcomes.

## Methods

A literature search was performed in Pubmed for English language studies spanning from 1984 to 2021. Phrases used in the search included, but were not limited to, “sleep,” “sleep disturbance,” “circadian rhythm,” “female reproductive health,” “menarche,” “fertility,” and “pregnancy.” These articles were assessed for relevance and quality. Articles were selected as relevant if they were: practice guidelines, retrospective cohort studies, observational studies, randomized control trials, prospective studies, literature reviews, systematic reviews, or meta-analyses that addressed sleep- or circadian rhythm-related molecular genetic pathways or addressed the relationship between one or more sleep parameters and reproductive health in women. Studies were excluded if they were case reports, editorials, or abstracts. Studies in rodent models were included only with respect to discussion of molecular pathways, as studies in humans are lacking.

### Molecular genetic pathways within the brain

In this section we review the molecular genetic pathways by which circadian rhythm regulates reproductive physiology. To discuss how alterations and/or mutations in such molecular pathways impact reproductive health, we reference rodent studies that may inform the human situation.

**Fig. 1:**
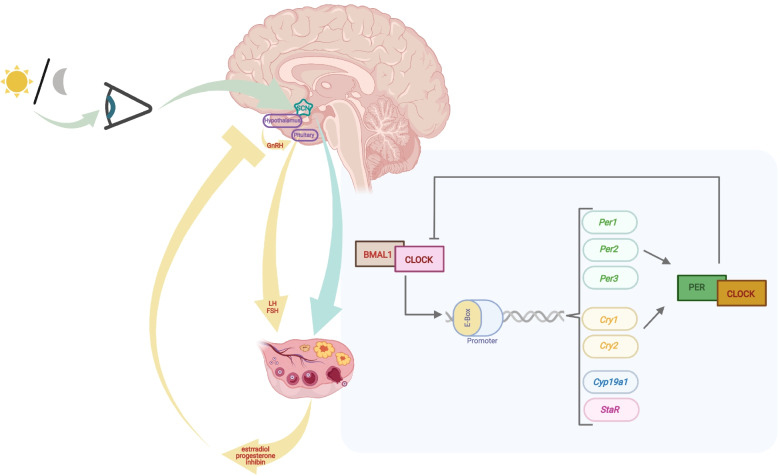
Organization of the circadian clock within the hypothalamic–pituitary–ovarian axis. Afferent neuronal tracts of the photosensitive ganglion cells of the retina convey light–dark input to the SCN, composing the endogenous molecular clock. The circadian clock works at the HPG axis by means of transcription and translation feedback loops to convey temporal signals to organs and tissues to carry out vital homeostatic functions. The *Clock*-*Bmal1* dimer activates the transcription of *Clock*-related genes, the products of which mediate various reproductive processes

Physiological and behavioral processes are rhythmically controlled by an endogenous molecular clock within the suprachiasmatic nucleus (SCN). Afferent neuronal tracts, such as those originating from the photosensitive ganglion cells of the retina, travel to the SCN to synchronize circadian oscillators with environmental cues, generating autonomous oscillations with an approximately 24-h period, known as circadian rhythms (Fig. 1). As such, zeitgebers (external cues that synchronize biological rhythms), including light–dark cycles, entrain the circadian clock, ensuring its synchrony with the solar day [18]. Exposure to erroneous zeitgebers, whether it be due to sleep deprivation, disruption, or dysregulation, may disrupt circadian homeostasis and lead to detrimental sequelae [19].

The circadian clock constitutes an autoregulatory succession of expression, accumulation, and degradation of Clock gene products via transcription and translation feedback loops. This autonomous molecular oscillator conveys temporal signals to organs and tissues to carry out vital homeostatic functions. Clock genes including *Bmal1, Clock, Per1, Per2, Per3, Cry1, Cry2, Dec1, Dec2,* and their molecular products have demonstrated importance for regulation of female reproductive processes (Fig. 1) [20]. *Bmal1* and *Clock* form a dimer and regulate *Per1, Per2, Cry1* and *Cry2* expression by binding to the E-box region of their promoters (Fig. 1) [20]. *Per1, Per2, Cry1* and *Cry2* form a protein complex, providing negative feedback on transcription regulation of *Bmal1*-*Clock* dimer (Fig. 1).

Reproductive hormones demonstrate circadian rhythmicity and are synchronized by the SCN [21]. Proper SCN rhythmicity, mediated by Clock genes, stimulates Gonadotropin Releasing Hormone (GnRH) pulsatility and pituitary luteinizing hormone (LH) release through two different neural networks: vasoactive intestinal peptide (VIP)-containing neurons and arginine vasopressin (AVP)-containing neurons [18]. VIP-containing neurons project from the SCN to GnRH neurons directly, whereas AVP-containing neurons project from the SCN to anteroventral periventricular nucleus Kisspeptin neurons and stimulate GnRH and LH secretion indirectly [22–24]. *Clock* mutant mice exhibit decreased expression of AVP in their SCN and have irregular estrous cycles without an LH surge [25]. Interestingly, supplementation of AVP in mice can induce an LH surge to some extent [25].

Daily oscillations in Clock genes serve regulatory functions in ovarian tissue, particularly within the follicle: theca cells, granulosa cells, and oocytes [26, 27]. In theca cells, androgenesis through Cyp17a1 expression is altered in *Bmal1* knockout mice [28]. Female mice lacking *Bmal1* also do not exhibit any LH or follicle stimulating hormone (FSH) surge [29]. Within granulosa cells, periodic fluctuations in estradiol and progesterone have been observed and are mediated via circadian regulation of aromatase [24]. The *Bmal1*-*Clock* dimer binds to the E-box sites of *Cyp19a1* and *StAR* promoter, regulating their transcription and the synthesis of estrogen and progesterone [30, 31]. Estrogen levels in turn may synchronize circadian rhythm by regulating *Per1* and *Per2* expression [32, 33] and modulating neuronal activity in the SCN via estrogen receptors (ER) [34]. Furthermore, *Clock* gene can modulate ER alpha transcriptional activity [35].

Fine circadian rhythm regulation is not only important for maintaining the complex and time sensitive pathways of reproductive hormone synthesis and secretion, but also reproductive processes including folliculogenesis, ovulation, fertilization, and implantation [36–39]. Sleep deprivation negatively affects fertility through alterations in expression of *Clock* and *Clock*-related genes [18]. Studies carried out on murine *Clock* gene knockout models conclude that circadian rhythm disorders may impact fertility, independent of the hypothalamic-pituitary axis [7]. For example, *Clock* and *Bmal1* deficient mice exhibit ovulatory dysfunction and decreased fertility [36, 40], while *Per* and *Cry* mutations in female rodents lead to reduced reproductive rates and litter size [31]. In a study employing a transgenic mouse model of the *Clock* gene, characterized by a defective form of *Clock* protein, able to produce the *Bmal1*-*Clock* dimer, but unable to regulate *Per* and *Cry* transcription, affected mice exhibit loss of circadian rhythmicity as well as a higher rate of pregnancy failure [41, 42]. Expression levels of Clock genes in various stages of folliculogenesis have also been demonstrated. In mice, immature germinal vesicle stage oocytes express more *Bmal1, Clock*, *Per1, and Cry1* compared to mature metaphase II stage oocytes [43, 44].

Clock genes also regulate endometrial decidualization, and thus implantation. In mice, absence of *Bmal1* leads to delayed puberty, decreased progesterone levels, irregular estrous cycles, small ovaries and uterus, and increased implantation failure [40, 45]. In humans, progesterone acts as promoter of *Per1* transcription, which in turn increases endometrial decidualization [46]. Furthermore, in a case control study of 268 women with at least three idiopathic miscarriages and 284 women with at least two live births and no history of pathologic pregnancies, polymorphisms of Clock genes were detected at higher concentrations in the peripheral blood of women with recurrent pregnancy losses [47].

Disruption of *Clock* expression may accelerate reproductive aging and lead to POI. *Per1*-*Per2* deficient mice have premature decline in ovarian reserve, irregular estrous cycles, and decreased reproductive rates [31, 39]. Another study demonstrates that the ovarian aging phenomenon can be recovered with tuning of light–dark cycles resembling the endogenous rhythm in *Cry* knockout female rodents [48].

### Sleep and reproductive endocrinology

Synthesis, secretion, and metabolism of hormones are in synchrony with the circadian rhythm and regulated by sleep patterns [6, 49]. For example, gonadotropins, sex steroids, and sex hormone binding globin (SHBG) exhibit diurnal rhythms in reproductive age women [16, 21, 50–54]. In the follicular phase of the menstrual cycle, FSH, LH, estrogen, progesterone, and SHBG display rhythmicity, whereas in the luteal phase, only FSH and SHBG demonstrate rhythmicity [21]. Disruption of SCN rhythmicity, as may occur with altered sleep, leads to disruption of the hypothalamic-pituitary–gonadal (HPG) axis, and thus loss of synchronized release of reproductive hormones [18], which may contribute to altered reproductive processes [24, 55–57].

In the following section we review the relation between sleep and female reproductive endocrinology. We explore the unique physiologic rhythms of reproductive and reproductive-related hormones as well as how alterations in sleep patterns may lead to impaired hormone production/secretion and endocrinopathies.

#### FSH

FSH stimulates growth of ovarian follicles and estrogen production by granulosa cells. FSH exhibits significant 24-h rhythms during both the follicular and luteal phase of the menstrual cycle [21]. A study conducted on 160 normally cycling reproductive-age women revealed a positive correlation between FSH and sleep duration, after adjusting for age and body mass index (BMI) [58]. Furthermore, FSH levels were 20% higher in long-time sleepers as compared to short-time sleepers [58]. On the other hand, a small study in 10 women, suggests that partial sleep deprivation does not change FSH levels in women during the early follicular stage of the menstrual cycle, though this may be because the study was not adequately powered [59]. In a cross-sectional study nested in the Study of Women's Health Across the Nation (SWAN), a longitudinal study of the menopausal transition, involving 365 middle-aged women, longer sleep duration as measured by in-home polysomnography and poor sleep quality as reported by questionnaire were associated with more rapid rate in FSH change, which typically indicates menopausal transition [60].

#### LH

LH regulates production of androgens from theca cells and estradiol from granulosa cells, ovulation, and the release of progesterone after ovulation by the corpus luteum. Like FSH, LH also exhibits significant 24-h rhythms during the follicular phase of the menstrual cycle [21]. In an observational study of 11 women, interpulse intervals of LH were longer and pulse amplitude greater during sleep periods in the early follicular phase regardless of time of day [61], implying that sleep is associated with a decrease in GnRH pulse frequency. During periods of sleep, LH pulses occurred most commonly in association with brief awakenings, suggesting that interruptions from sleep allow escape from the inhibitory effect of sleep on pulsatile GnRH secretion [61]. This is corroborated by evidence that indicates that LH concentrations increase during partial sleep deprivation [59]. A separate interaction of time of day on LH pulse dynamics has also been reported: during sleep, mean LH levels were lower at night, but not during the day; in the absence of sleep, mean LH levels and LH pulse amplitude were greatest in the evening [61].

#### Estradiol

Estradiol, secreted by granulosa cells of ovarian follicles, is the primary estrogen during reproductive years, playing an important role in ovulation, follicular growth, and development, as well as in maintenance of female sex characteristics. In a study of 95 regularly menstruating women, women with regular sleep schedules exhibited 60% lower levels of estradiol, when compared to women with more irregular schedules [62]. The same study found no association between sleep duration and estradiol levels [62]. However, in a prospective study of 259 regularly menstruating women, mean estradiol concentrations significantly increased by 3.9% for every additional hour of daily sleep [63]. In the same study, women with morning chronotypes had earlier rises in estradiol during their cycles [63]. In a small study of 10 women, estradiol concentrations increased during partial sleep deprivation [59]. Furthermore, in a cross-sectional study among 365 women, estradiol levels were negatively associated with sleep quality [60].

#### Progesterone

Progesterone regulates the uterine lining and is essential for implantation and maintenance of pregnancy. In a prospective study of 259 regularly menstruating women, for every hour increase in daily sleep duration, mean luteal phase progesterone levels increased by 9.4% [63]. Interestingly, stress is associated with decreased levels of progesterone [17], which may imply that sleep, a physiological stressor, is also associated with lower levels of progesterone. Unfortunately, these is otherwise a paucity of evidence on the impact of sleep parameters on endogenous progesterone levels.

Numerous studies indicate that progesterone acts as a sleep inducer, anxiolytic, and potent respiratory stimulant [64, 65]. Progesterone administration shortens non-REM sleep and prolongs REM sleep duration, likely due to the sedative effects exerted by its metabolite [66]. In women with PCOS, low levels of progesterone are associated with increased sleep-disordered breathing [67], whereas high levels of progesterone are associated with lower rates of obstructive sleep apnea (OSA) and obesity-related hypoventilation [65]. Moreover, the loss of progesterone during menopause is linked to sleep complaints [64]; and administration of intranasal progesterone in postmenopausal women has sleep promoting effects [68].

#### Thyroid stimulating hormone (TSH)

Elevated levels of TSH are associated with anovulation, menstrual irregularities, amenorrhea, and recurrent miscarriage [69]. TSH exhibits a rhythmic pattern: TSH increases prior to sleep onset and continues to increase over the course of the sleep period/night when it peaks; TSH subsequently decreases during the wake period/day [70]. Sleep deprivation has shown inconsistent effects on TSH secretion. While acute, extreme deprivation increases the secretion of TSH and augments the surge of TSH release, chronic, modest sleep deprivation suppresses the secretion of TSH [59, 71–74]. Unfortunately, several of these studies were conducted solely in men. For example, in a cohort of 11 young men, acute sleep deprivation was associated with a near twofold rise in TSH levels, suggesting that sleep has an inhibitory effect on TSH release [70]. A study among both women and men with major depression found insomnia ratings to be negatively correlated with TSH levels [75]. In a study utilizing the Korea National Health and Nutrition Examination Survey, both shorter and longer sleep durations were associated with an increase in the risk of subclinical thyroid dysfunction compared to optimal sleep duration [76].

#### Prolactin (PRL)

PRL stimulates lactation in women and has pleotropic actions. Prolactin surges upon sleep onset regardless of the time of day [6, 77, 78]. Decreased dopaminergic inhibition of PRL during sleep is likely to be the primary mechanism underlying nocturnal PRL elevation [79]. Transient awakening inhibits PRL secretion and final awakening corresponds to a rapid offset of PRL secretion [6, 80]. As such, PRL levels are suppressed among those with sleep deprivation or disruption [59, 81]. Women with narcolepsy and OSA on CPAP have lower levels of sleep related PRL [78, 82]. Interestingly, in a study of 55 women with infertility and endometriosis, nocturnal patterns of PRL secretion were altered and proposed as an etiology for sub/infertility among women with endometriosis [83]. Hypnotics including benzodiazepine, imidazopyridine, triazolam and zolpidem, as well as ramelteon (melatonin receptor agonist) cause an increase in nocturnal PRL rise, resulting in concentrations near pathological range for part of the night [77, 84]. In a study restricted to men, sleep quality appears not to affect PRL secretory profiles [80]. Unfortunately, to our knowledge, no comparable studies regarding sleep quality were conducted in women. However, a study among 56 Swedish women reveals an association between perceived job strain and an elevation in plasma PRL after adjusting for confounders [85]. As sleep disturbance and stress are highly correlated, this study implies that sleep disturbance may theoretically be associated with higher levels of plasma PRL, an etiology of infertility.

#### Glucocorticoids

Glucocorticoids have numerous functions for bodily homeostasis [86], and are released in a diurnal pattern with peak levels linked to the start of the activity phase [87]. Stressful stimuli increase the activation of the hypothalamic-pituitary axis, increasing production of corticosterone from the adrenal glands as well as altering gonadotropin levels [86, 88]. Glucocorticoids are the final mediators in hypothalamic–pituitary–adrenal axis and critical for the pathogenesis of sustained stress-related sleep disorders [89]. Glucocorticoids may regulate sleep directly via corticosteroid receptors in the brain [90, 91]. Moreover, sustained stress increases cortisol levels and may induce sleep disorders [92], including impaired sleep quality and shortened sleep duration [89].

Glucocorticoids indirectly affect ovarian function by altering levels of gonadotropins, metabolic hormones, and growth factor, as well as inhibiting Kisspeptin neurons and gonadotropic inhibitory hormone [18]. Glucocorticoids also regulate many of the signal transduction and biological processes important for reproductive capacity via tissue-specific glucocorticoid receptors in the uterus, ovaries, and mammary glands [86]. Additionally, differential expression of enzymes that regulate the activation and inactivation of glucocorticoids in the ovary allows for local, intracellular regulation of glucocorticoid metabolism [93, 94].

Stress generates glucocorticoid-mediated adverse effects on reproductive physiology [86]. For example, in prepubertal girls, glucocorticoid secretion is associated with age of puberty onset [95]. Stress-induced levels of glucocorticoids are also associated with reduced fecundity [86, 96, 97]. High perceived stress during pregnancy is a risk factor for preterm labor and poor outcomes for offspring [98, 99]. Increased stress is also associated with poorer ART outcomes (less oocytes retrieved and lower fertilization, pregnancy, and live birth rates) [100–102], though results are inconsistent [103, 104]. Importantly, the association may be bidirectional such that psychological stress may impact reproductive potential and/or impaired reproductive function may worsen psychological distress [105, 106]. Moreover, stress may induce or exacerbate sleep disturbance and vice versa; and both may independently or collectively impact reproduction.

#### Melatonin

Melatonin is primarily produced in the pineal gland. It plays a key role in synchronizing the circadian sleep pattern [107] and possesses multifunctional bioactivities, including anti-oxidative [108], anti-inflammatory, anti-apoptotic, endocrinologic, and behavioral [109]. Studies confirm that melatonin is also produced in the peripheral reproductive system (granulosa and placental cells) and acts as a modulator of ovarian and uterine function [18, 110, 111]. Through melatonin receptor-mediated actions at the level of granulosa-luteal cells, melatonin regulates progesterone production, GnRH secretion, and LH and GnRH receptor gene expression [110].

Altered sleep patterns deregulate the endogenous secretion of melatonin and may impair reproductive health. For example, high melatonin levels are associated with delayed puberty and impaired ovulation, whereas low melatonin levels are associated with precocious puberty [112–116]. Moreover, melatonin in ovarian follicles is thought to protect oocytes against oxidative stress [117]. Sleep deprivation reduces the secretion of endogenous melatonin thereby limiting follicular melatonin levels and exposing follicles to reactive oxygen species [118, 119], thus reducing oocyte quality and quantity as measured in infertile women [118, 119]. In like manner, follicular melatonin levels were observed to be significantly lower among women with idiopathic infertility and associated with marked oxidative imbalance [120]. Among 63 women undergoing in vitro fertilization (IVF), follicular melatonin was positively correlated with markers of ovarian reserve (antral follicle count (AFC), and anti-mullerian hormone (AMH) levels), number of retrieved oocytes, total fertilized oocytes, normally fertilized oocytes, cleaved oocytes, number of high-quality day three embryos, blastocysts obtained, and total embryos obtained [121]. Melatonin is also proposed to serve a role in ovarian aging [122], and studies suggest that melatonin supplementation confers protective effects against POI and may be used as a potential treatment modality [123]. In addition, melatonin is involved in uterine homeostasis, decidualization/implantation, and placentation [124]. Irregular production and low circulating levels of melatonin are associated with low implantation rates, recurrent miscarriage, and premature birth [124]. Melatonin, which passes the placental barrier, is also responsible for advancing the maturation of the fetal SCN and reducing fetal oxidative stress [111].

Given the associations between melatonin and parameters of reproductive function, numerous studies sought out to evaluate the potential therapeutic effects of melatonin supplementation. Growing evidence indicates that melatonin supplementation ameliorates intrafollicular oxidative balance [120] and improves ART outcomes, specifically the number of oocytes retrieved, oocyte quality and maturation [109, 120, 125], rate of fertilization [118, 125], embryo quality [111, 118, 125], and pregnancy and live birth rates [120]. This is in contrast to data that suggests that excess melatonin is associated with amenorrhea and infertility [126–129].

While the studies above suggest that melatonin may improve fertility parameters, melatonin supplementation does not necessarily improve impaired sleep among women with infertility. In a double-blinded placebo controlled randomized control study of 116 women, those undergoing IVF had no dose–response effect of melatonin on objective measures of sleep quantity or quality, though the study may not have been powered adequately to detect statistically significant differences between groups [130].

### Sleep and clinical reproductive outcomes

In this section we review the association between sleep parameters and clinical reproductive outcomes, including puberty, ovarian function, fertility, ART, and pregnancy.

#### Reproductive development/puberty

Puberty is a process of progressive developmental change by which children mature to achieve adult reproductive function and potential. Adolescence and puberty are accompanied by changes in sleep–wake patterns, suggesting that reproductive maturation may be a critical stage in the development of sleep habits [131]. Pubertal youth experience delayed sleep phase (the natural tendency for later bedtime), longer sleep latency, less deep sleep, and a greater tolerance to stay up compared to pre-pubertal youth [132]. In a Chinese cohort study, older age at menarche was associated with longer sleep duration (OR = 1.11; 95% CI = 1.01–1.21), though other measures of puberty timing (breast, genitalia, and pubic hair development) were unrelated to sleep duration [133]. Another study found that pubertal maturation (measured by Tanner stages 1 to 5) was associated with a progressive increase in the prevalence of insomnia symptoms, which more significantly affects girls: 3.4–12.2% in girls (3.6-fold) compared to 4.3–9.1% in boys (2.1-fold) [134]. This is corroborated by a study which reports that the onset of menses is accompanied by a 2.75-fold increase in risk of insomnia [135]. In a cohort of 5,800 Chinese female adolescents, age at menarche ≤ 11 years was significantly associated with insomnia symptoms in 12- to 14-year-old girls [136]. In a longitudinal prospective study, earlier pubertal timing in girls was associated with shorter sleep duration [132]. Moreover, among black adolescents, earlier pubertal timing was associated with later bedtime, which was not significant among other racial/ethnic groups [132]. These studies collectively suggests that reproductive developmental changes in girls are accompanied by physio-psychological stressors that may induce insomnia, a phenomenon that may be further exacerbated by earlier pubertal timing and racial/ethnic disparities.

International, historical trends demonstrate a progressive decrease in age at puberty, likely as a result of environmental exposures [137]. Obesity, endocrine disrupting chemicals, and stress are among some factors associated with earlier age at puberty [137]. Early puberty is linked to breast cancer, heart disease, diabetes, and all-cause mortality [138]. While the studies presented above suggest that reproductive maturation may alter sleep patterns, we identified no studies addressing the reverse relationship. Theoretically, sleep may induce stress-mediated changes in pubertal timing. As such, further studies are required to investigate the impact of sleep parameters on reproductive development in girls.

#### Ovarian function

##### Ovulation and menstruation

According to the National Institutes of Health (NIH), a typical menstrual cycle lasts between 21–35 days with median cycle duration of 28 days and bleeding duration of two to six days. The results of a meta-analysis suggest that disturbed sleep is associated with a 46% increased risk of menstrual irregularity [15]. A cross-sectional study of 801 adolescents found that prevalence of self-reported menstrual cycle irregularity increased with decreasing sleep duration [139]. In a multivariable logistic regression analysis, the odds ratio (OR) of menstrual cycle irregularity increased with shorter sleep duration (OR [≤ 5 h sleep] 2.36, 95% CI = 1.02–5.47; OR [6-7 h sleep] 1.51, 95% CI = 0.81–2.82) after adjusting for potential confounders [139]. Among a large sample of Chinese adolescent girls, insomnia symptoms were associated with a 46% increase in risk of irregular periods, 99% increase in risk of period pain, and 21% increase in risk of menstrual flow length ≥ 7 days; meanwhile, poor sleep quality was associated with a 72% increase in risk of irregular periods and 78% increase in risk of period pain [136]. Circadian dysrhythmia is also associated with menstrual cycle dysfunction. In a meta-analysis of 16 female cohorts (*N* = 123,403), women who engaged in shift work had a 22% increase in risk of menstrual cycle disruption [140]. Among a national cohort study of 71,077 nurses, rotating shift work was associated with increased risk of menstrual irregularity (defined as 7 day variability from cycle to cycle): relative risk (RR) 1.13 for 1–9 months of rotating shifts, RR 1.18 for 10–19 months, and RR 1.23 for 20 + months [141]. Those who worked 20 + months of rotating shifts had an increased risk of menstrual cycles lasting more than 40 days (RR 1.49, 95% CI = 1.19–1.87), and there appeared to be a dose response [141]. However, since the current work status or time since last month of rotating shift work was not reported, it is unclear whether the association is a cumulative effect of shift work over the two-year study period, or whether it is a short-term, reversible effect among those working shifts more frequently and/or recently [141]. Furthermore, the study did not assess permanent night work or other sleep behaviors, which may potentially differ among groups [141]. In a cross-sectional study, 53% (36/68) of nurses noted menstrual changes (cycle length, menstrual flow, menstrual pain, and duration of menses) when working shiftwork [142]. Nurses noting menstrual changes also reported more physiological symptoms, sleeping approximately one hour less when working nights, and lengthened time to fall asleep when working nights [142]. In a prospective study, nurses working in high stress units exhibited a greater than 4- to fivefold risk of long and monophasic cycles [143]. Likewise, in a cohort study, nurses who worked rotating shifts or in emergent care units/wards, presumably higher stress environments, had a significantly higher prevalence of irregular ovarian cycle patterns [144]. Moreover, in a survey of 1,458 female nurses, job stress was associated with a threefold increase in amenorrhea and other menstrual abnormalities [145].

##### Polycystic Ovarian Syndrome (PCOS)

PCOS is a condition characterized by chronic anovulation, oligo/amenorrhea, clinical or biochemical hyperandrogenism, and/or polycystic ovaries [146]. Women diagnosed with PCOS are at higher risk of cardiovascular disease, diabetes, obesity, depression and anxiety [146]. Women with PCOS also more commonly report sleep disturbances [147], are twice as likely to report difficulty achieving and maintaining sleep [148], and are 30 times more likely to suffer from sleep disordered breathing [149]. OSA is prevalent in 44% of obese women with PCOS, compared to 6% of age- and weight-matched reproductively normal women [150]. A meta-analysis of eight studies in adults and five studies in adolescents reported a nearly 10-times increase in risk of OSA in adult patients with PCOS, though the risk was not significantly increased in adolescents [151]. Among 1,603 infertile women enrolled in two concurrent randomized clinical trials, women with PCOS more commonly experienced sleep duration less than six hours, habitual snoring, and clinical sleepiness compared to women with unexplained infertility [147]. Nevertheless, clinical symptoms of OSA and short sleep duration did not affect fertility treatment response [147].

The association between sleep disturbances/disorders and PCOS is thought to be mediated in part by hyperandrogenemia, insulin resistance, melatonin, and/or psychosocial sequelae (depression and anxiety) [152]. For example, in a polysomnographic study comparing women with and without PCOS, insulin resistance was the strongest risk factor for sleep apnea, after controlling for age, BMI, and testosterone levels [149]. Moreover, insulin resistance and glucose intolerance were highly correlated with the presence and severity of OSA in women with PCOS [153]. Adolescent girls with PCOS treated with metformin reported reduced sleep disturbances and daytime sleepiness, though the improved sleep parameters may be due to concomitant reductions in BMI and hyperandrogenemia [154]. Furthermore, apnea–hypopnea indexes in women with PCOS correlate with serum and unbound testosterone levels [150, 155]. Melatonin rhythms are also disrupted in women with PCOS [152]: women with PCOS exhibit higher levels of melatonin in serum and urine [156, 157], yet lower concentrations in follicular fluid [119, 158].

While sleep pathologies are highly prevalent in women with PCOS, only one study to our knowledge investigated whether sleep behaviors contribute to increased risk of developing PCOS and PCOS-related metabolic abnormalities (insulin resistance and decreases glucose tolerance). In a large population-based multicenter survey, after adjusting for confounders, long-term rotating shift work was associated with an 80% increased risk of PCOS, suggesting that circadian dysrhythmia may be a risk factor for PCOS [159].

##### Premature ovarian insufficiency (POI) 

POI is defined as the cessation of menses due to ovarian failure before the age 40 years. Premature declining ovarian reserve is replicated in multiple *Clock*-gene deficient rodent models, such as *Per1* and *Per2* loss-of-function mutations, suggesting that disruption in circadian rhythmicity may play a role in the pathophysiology of POI [24, 39]. In a cross-sectional study of 61 women with POI receiving hormone therapy (HT) compared to 61 age-matched individuals, women with POI receiving HT had poorer sleep quality, took longer to fall asleep, and had higher fatigue index [160]. The directionality of these associations remains uncertain. Unfortunately, there is otherwise limited research on the association between altered sleep and POI.

#### Fertility

Infertility is defined by the inability to conceive within 12 months for women under 35 years of age and 6 months for women 35 years or older. Women with infertility report high rates of sleep disruption and poor sleep quality, associations that are likely bidirectional [9–11]. Among 1,176 couples followed every eight weeks for two years, couples whose female partners reported trouble sleeping more than 50% of the time had reduced fecundity when compared to women who reported no trouble sleeping, though not statistically significant with fecundability ratio of 0.83 (95% CI 0.7–1.00) [161]. Within the same cohort, couples whose male partners slept less than six hours a night had significantly reduced fecundity when compared to men who slept eight hours or more a night, with fecundability ratio of 0.68 (95% CI 0.5–0.93) after adjusting for confounders including but not limited to age, BMI, partner's sleep duration intercourse frequency, and number of male parameters [162]. Interestingly, these findings persisted among couples attempting to conceive for less than three cycles at study entry, among whom reverse causation is less likely (i.e., subfertility causing disturbances in sleep) [162]. In a study gauging responses to mailed questionnaires to 3,985 members of the Swedish Midwives Association with an 84% response rate, midwives who worked rotating two-shift, three-shift, or only night shifts had reduced fertility compared to those working only in the daytime, with fecundability ratios of 0.78 (95% CI 0.65–0.94), 0.77 (95% CI 0.60–0.98), and 0.82 (95% CI 0.64–1.03), respectively, after adjustment for covariates [163]. In a multinational European study, shift work was also associated with subfecundity [164]. In sum, this evidence strongly supports the hypothesis that sleep affects natural fertility potential.

#### Assisted reproductive technology (ART)

##### Intrauterine insemination (IUI)

The potential association between sleep and IUI cycles has been scantily studied. In a cross-sectional study of 117 women undergoing IUI, 35% of women reported sleep disturbances [9]. While this study reveals an association between IUI and sleep disturbance, it remains unclear whether disturbed sleep is a risk factor for necessitating IUI or whether disturbed sleep impacts IUI outcomes [9].

##### In vitro fertilization (IVF)

Among a sample of 100 women, sleep disturbance was recognized as the most significant psychological stressor experienced by infertile women undergoing IVF [10]. Sleep patterns in women undergoing IVF are therefore an important area of research [10], yet available studies are limited. Among the few studies conducted, evidence suggests that altered sleep patterns are associated with poorer IVF outcomes. For example, in a prospective cohort study of 431 day-shift workers and 42 evening/night/rotating-shift workers, women who worked evening/night/rotating shifts had 2.3 fewer mature oocytes retrieved on average, compared with women who worked day-only shifts after adjusting for age, BMI, education, and infertility diagnosis [165]. In another prospective cohort study, women with recurrent implantation failure slept on average 53 min less than fertile women without endometrial pathology [166]. Though this study demonstrates an objective observation of sleep time reduction, as measured by actigraphy, it does not address causality [166]. In a pilot prospective cohort study of women undergoing IVF, the expected number of oocytes retrieved increased by 1.5 for every 1-h increase in total sleep time (adjusting for AMH and day-3 FSH) with a trend towards statistical significance (*p* = 0.09). Interestingly, this study assessed sleep using multiple modalities, including actigraphy and several verified questionnaires. In a regression analysis, AMH, day-3 FSH, and baseline total sleep time accounted for 40% of the observed variance in oocytes retrieved (adjusted R^2^ = 0.40, *p* = 0.03) [106]. Especially as IVF remains limited in efficacy, yielding live births in less than 40% of cycles [167], the association between sleep alterations and IVF parameters and outcomes warrants further investigation.

#### Pregnancy outcomes

Psychophysiological changes caused by pregnancy are believed to account for the high incidence of sleep disturbance and extreme sleep duration (long or short sleep durations) observed among pregnant women [168–170]. Longitudinal assessments of sleep using polysomnography report that total sleep time at night increases in the first trimester, but decreases by the third trimester [171]. In contrast, in a large survey-based study of 2,427 women, the frequency of women who reported ≤ 6 h of total sleep (naps plus nocturnal sleep) increased from approximately 17% in the first trimester to 33% in late pregnancy, suggesting that the proportion of women with insufficient sleep increases through pregnancy [172]. Approximately 28% of pregnant women sleep less than seven hours a night and nearly 3% sleep more than nine hours a night [168]. In this section we review the associations between sleep parameters and adverse pregnancy outcomes, including pregnancy loss, low birth weight, gestational diabetes (GDM), hypertensive disorders, preterm birth, and delivery method. Although studies suggest that certain sleep disturbances are related to adverse pregnancy outcomes, small sample sizes and cross-sectional designs preclude clear conclusions [173].

##### Early pregnancy loss

Early pregnancy loss (a term used interchangeably with miscarriage and spontaneous abortion) is defined as a nonviable, intrauterine pregnancy diagnosed within the first 12 6/7 weeks of gestation [174]. Early pregnancy loss affects 10% of clinically recognized pregnancies [174]. Known risk factors for early pregnancy loss include advanced maternal age and prior miscarriage [174]. Some evidence also supports the association between altered sleep parameters and increased risk of miscarriage. In a prospective cohort study, women with recurrent miscarriage slept 36 min less per night than women in the control group as measured via actigraphy [166]. In a case control study, sleeping ≤ 8 h a day was associated with a 3.8-fold increase (95% CI:1.01–14.3) in risk of miscarriage after controlling for period of gestation [175]. In a study of 2,000 female flight attendants, when the sleep period overlapped with work times based on the time zone, female flight attendants had significantly higher risk of miscarriage [176]. In a meta-analysis night shift workers had a 41% increased adjusted risk of early pregnancy loss after adjusting for confounders, though shift work was not associated with significantly higher risk of early pregnancy loss [140]. Similarly, an epidemiologic study found the risk of pregnancy loss to be four-times higher among women with fixed evening work schedules (three or four o’clock to eleven or twelve o’clock) and more than double among women with fixed night schedules when compared to fixed day schedules [177]. In combination, these studies suggest that shorter sleep duration and circadian rhythm disruption may be a modifiable risk factor for early pregnancy loss.

##### Gestational diabetes (GDM)

GDM refers to the development of glucose intolerance during pregnancy [178]. GDM is associated with numerous maternal and fetal complications [178]. Growing evidence indicates that sleep alterations alters glucose metabolism and increases risk of insulin resistance and diabetes, via increased levels of oxidative stress, inflammation, sympathetic activity, and cortisol [179]. A meta-analysis of seven studies assessing the relationship between sleep duration during pregnancy and GDM development, reported that extreme sleep duration (long or short sleep duration) during early and middle pregnancy was associated with a 1.35-fold increased risk of GDM [180]. Moreover, long sleep duration during early and middle pregnancy was positively associated with incident of GDM [180]. Among this meta-analysis was a study conducted in China including 12,506 women, 919 (7.3%) of whom had GDM [181]. When comparing pregnant women who slept ≥ 9 h/day (55%) or < 7 h/day (2%) to women who slept 7–9 h/day (43%), those who slept ≥ 9 h had increased risk of GDM (OR 1.21; 95% CI = 1.03–1.42), while those who slept < 7 h had no significant increased risk (OR 1.36; 95% CI = 0.87–2.14) [181]. Pregnant women who reported moderate (59.9%) and poor sleep quality (2.2%) had increased risk GDM (OR 1.19, 95% CI = 1.01–1.41 and OR 1.61; 95% CI = 1.04–2.50, respectively), when compared to pregnant women who reported good sleep quality (37.9%) [181]. In a meta-analysis of 16 studies, including 2,551,017 pregnant women and 142,103 GDM cases, both short and long sleep duration were associated with increased risk of GDM (RR [short] 2.02, 95% CI = 1.31–3.11; RR [long] 1.19, 95% CI = 1.04–1.35) [182]. Moreover, poor sleep quality, snoring, and OSA were also associated with increased risk of GDM (RR [poor sleep quality] 1.26, 95% CI = 1.11–1.44; RR [snoring] 1.45, 95% CI = 1.12–1.87; and RR [OSA] 1.60, 95% CI = 1.21–2.12) [182]. Within this meta-analysis, a study conducted by Zhong et al., in 4,066 singleton pregnancies, reported an increased risk of GDM among women with poor sleep quality during early pregnancy (OR 1.77, 95% CI = 1.20–2.61), a finding that was more robust among women ≥ 30 years of age (OR 2.35, 95% CI = 1.35–4.09), with a family history of diabetes (OR 4.02, 95% CI = 1.54–10.48), or with concomitant longer nighttime sleep duration (OR 2.27, 95% CI = 1.20–4.29) [183].

Large prospective cohort and population-based studies also associate sleep disordered breathing with higher odds of GDM [184, 185]. In a case–control study of 46 women with newly diagnosed GDM and 46 healthy control subjects, matched for age, gestational age, body mass index, race, and parity, women with OSA had a higher GDM risk (OR 4.71; 95% CI = 1.05–21.04) [186]. GDM risk was also significantly higher among women with higher overall apnea–hypopnea index, higher apnea–hypopnea index in REM, and higher oxygen desaturation index, as measured by polysomnography as well as symptom scores [186]. Furthermore, severity of sleep disordered breathing in women with GDM correlates with higher nocturnal and morning glucose levels, after adjusting for BMI and medications [187], suggesting that sleep disordered breathing may worsen blood glucose control in women with GDM.

##### Hypertensive disorders of pregnancy

Hypertensive disorders of pregnancy include chronic hypertension (occurring prior to 20 weeks’ gestation or persisting 12 weeks after delivery), gestational hypertension (occurring after 20 weeks’ gestation), and preeclampsia [188]. Preeclampsia is defined as hypertension with proteinuria, thrombocytopenia, renal insufficiency, impaired liver function, pulmonary edema, and/or cerebral or visual symptoms [188]. Hypertensive disorders of pregnancy are associated with increased neonatal and maternal morbidity and mortality [188]. Sleep disordered breathing and snoring are accompanied by an increased risk for gestational hypertension and preeclampsia [189–191]. In a study of 502 pregnant women, hypertension developed in 14% of snoring women, compared with 6% of non-snorers (*p* < 0.01), and preeclampsia occurred in 10% of snorers, compared with 4% of non-snorers (*p* < 0.05) [189]. Habitual snoring was independently predictive of hypertension (OR 2.03; *p* < 0.05) after controlling for weight, age, and smoking [189]. In a study conducted among 456 women in Argentina, snoring and sleep apnea were independently associated with pregnancy-induced hypertension and pre-eclampsia (OR [snoring] 1.82, 95% CI = 1.16–2.84; OR [apnea] 8.00, 95% CI = 2.71–23.55), irrespective of BMI before pregnancy, weight gain during pregnancy, neck circumference, smoking, alcohol, and age [191]. In a study among 25 patients with preeclampsia, subjects exhibited markedly altered sleep architecture: increased percentage of time spent in slow-wave sleep, longer latency to REM, and reduced time spent in REM [192]. In another small cohort study, total movement time and total frequency of body movements in bed were significantly increased in the preeclamptic group, though subjective quality of sleep was comparable [193]. Overall, the available evidence indicates that sleep disordered breathing and altered sleep architecture are associated with hypertensive disorders of pregnancy, though further studies are required to assess for any degree of causality.

##### Low birth weight

Low birth weight is associated with numerous harmful neonatal outcomes, including but not limited to hypothermia, septicemia, necrotizing enterocolitis, intraventricular hemorrhage, and respiratory distress [194], and is therefore an important measure when examining pregnancy outcomes. In a survey of women, shift work was associated with a significantly higher risk of low birth weight (< 2500 g) after adjusting for confounders (OR 2.1, 95% CI = 1.1–4.1) [195]. Multiple other studies corroborate the association between shift work and low birth weight [196–198]. In a cohort of 502 women in Sweden, a diagnosis of small for gestational age was given to 7.1% of infants of snoring mothers, compared to 2.6% of remaining infants (*p* < 0.05); and habitual snoring was independently predictive of growth retardation (OR 3.45; *p* < 0.01) after controlling for maternal weight, age, and smoking [189]. Overall, shift work and sleep disordered breathing may contribute to impaired fetal growth.

##### Preterm birth and delivery method

Preterm birth is defined as parturition prior to 37 weeks’ gestation. Preterm births account for approximately 70% of neonatal deaths and 36% of infant deaths as well as 25–50% of cases of long-term neurologic impairment in children, including cerebral palsy [199]. In a survey of women, shift work was significantly associated with preterm birth (OR 2.0; 95% CI = 1.1–3.4) [195]. In a meta-analysis of 10 studies, short sleep duration and poor sleep quality were associated with an increased risk of preterm birth (RR [sleep duration] 1.23, 95% CI = 1.01–1.50; RR [sleep quality] 1.54, 95% CI = 1.18–2.01) [200]. These studies suggest that sleep may be an important target for improving delivery timing and thus neonatal outcomes.

To our knowledge only one study addresses the association between sleep and delivery method. In a prospective observational study of 131 pregnant women, women who slept less than six hours a night had longer labors and were more than four times more likely to have cesarean deliveries [201]. Additionally, women with severely disrupted sleep also had longer labors and were more than five times more likely to have cesarean deliveries [201]. Importantly, fatigue was unrelated to method of delivery, and therefore not considered to confound the results. Based on the limited available evidence, the authors recommended that pregnant women sleep at least eight hours a night, and that sleep quantity and quality be included in prenatal assessments as potential predictors of labor duration and delivery type [201].

## Conclusions

Sleep is essential to human health, and when altered is associated with impaired reproductive function and generally poorer reproductive outcomes. The mechanisms by which sleep affects the female reproductive axis are likely multifactorial. Genetically encoded molecular *Clock* and *Clock*-related genes, including *Bmal1, Per1, Per2, Per3, Cry1, Cry2, Dec1, Dec2,* are critical for maintaining the circadian rhythmicity of reproductive hormone production and secretion [20]. Disruption of circadian rhythmicity, as may occur with altered sleep, leads to impaired HPG axis function, and thus disturbed reproductive processes. Moreover, studies of knockout or mutated animal models characterize the molecular genetic mechanisms by which circadian rhythm disorders may impact reproductive capacity and accelerate reproductive aging [31, 36, 39, 40], findings that may inform the human condition.

Hormones involved in the HPG axis, including GnRH, gonadotropins, and sex steroids demonstrate rhythmicity, which when altered may disturb all facets of reproductive development, function, and outcomes [18]. PRL, TSH, glucocorticoids, and melatonin levels and secretion patterns are also correlated with sleep parameters, and when altered may have detrimental effects on human reproductive health. For example, stress-induced levels of glucocorticoids are linked to reduced fecundity and preterm labor [86, 98, 99]; and decreased levels of serum and follicular melatonin may affect oocyte quantity and quality and implantation, miscarriage, and preterm birth rates [121, 124]. In some studies, melatonin supplementation improves ART success [109, 111, 118, 125].

The effects of sleep patterns on reproductive development are not well studied. Puberty is clearly accompanied by changes in sleep patterns [131, 132], but whether differences in sleep patterns precede and are associated with changes in pubertal onset remains unclear.

With regards to ovarian function, several large population studies reveal significant associations between menstrual cycle irregularity and/or prolonged cycles and decreased sleep duration, insomnia symptoms, shift work, or disturbed sleep [15, 136, 139–145]. Disrupted sleep patterns may also be associated with PCOS and POI, though studies are limited. Women with PCOS more commonly experience sleep disturbance and disorders [147], an association that is thought to be mediated by hyperandrogenemia, insulin resistance, melatonin, and/or psychosocial sequelae (depression and anxiety) [152]. One study found that circadian rhythm disruption may be a potential risk factor for PCOS and PCOS-related metabolic sequelae [153, 159]. While women with POI exhibit poorer sleep quality, take longer to fall asleep, and have a higher fatigue index [160], there is otherwise little evidence regarding the relation between sleep and POI in humans.

Numerous studies report a high prevalence of sleep deprivation and disruption among women with sub/infertility or undergoing ART [9, 10]. While the association between altered sleep and infertility may be bidirectional, one study reported reduced fecundity among sleep deprived couples attempting to conceive for less than three cycles, for whom reverse causation would be unlikely [161]. The relation between sleep patterns and IUI are insufficiently studied. Among women undergoing IVF, shorter sleep duration and shift work were associated with retrieval of fewer mature oocytes [165] and higher rates of implantation failure [166]. While no causality is established, these studies imply that sleep may be an important area of future research with regards to improving IVF outcomes.

Data linking sleep alterations and adverse pregnancy outcomes are suggestive but limited. Several studies indicate that shorter sleep duration and shift and night work are associated with higher rates of miscarriage [140, 166, 176, 177]. Sleep disordered breathing is also linked to hypertensive disorders of pregnancy [189–191]. Long and potentially short sleep duration, poor sleep quality, and sleep disordered breathing are associated with increased GDM risk. Other studies associate shift work and sleep disordered breathing with lower birth weights [189, 195–198]. Short sleep duration and poor sleep quality were also associated with an increased risk of preterm birth [200], longer labors, and higher rates of cesarean deliveries [201]. Importantly, fatigue could not account for the trends observed [201].

Overall, pathologic sleep patterns are associated with numerous detrimental reproductive health outcomes in women that warrant further investigation. While causality is not well established, it is highly plausible that the physiological stressors accompanied by sleep deprivation, altered sleep–wake cycles, sleep disruption, and sleep disorders impair related molecular pathways and contribute to endocrinopathies, which significantly impact reproductive health. Importantly sleep may be a modifiable target to improve reproductive capacity and outcomes, and studies aimed at applying improved sleep hygiene, cognitive behavioral therapy, optimized sleep duration, or other interventions may glean further knowledge on this vastly relevant area of study.

## Data Availability

Not applicable.

## Abbreviations

AFC: Antral follicle count
ART: Assisted reproductive technology
CI: Confidence interval
COPD: Chronic obstructive pulmonary disease
CPAP: Continuous positive airway pressure
FSH: Follicle stimulating hormone
GnRH: Gonadotropin releasing hormone
HPG: Hypothalamic-pituitary–gonadal axis
IUI: Intrauterine insemination
IVF: In vitro fertilization
LH: Luteinizing hormone
NIH: National Institutes of Health
OR: Odds ratio
OSA: Obstructive sleep apnea
PCOS: Polycystic ovarian syndrome
POI: Premature ovarian insufficiency
PRL: Prolactin
REM: Rapid eye movement
TSH: Thyroid stimulating hormone
